# Optimizing Patient Selection for Interhospital Transfer and Endovascular Therapy in Acute Ischemic Stroke: Real-World Data From a Supraregional, Hub-and-Spoke Neurovascular Network in Germany

**DOI:** 10.3389/fneur.2020.600917

**Published:** 2020-12-04

**Authors:** Maria-Ioanna Stefanou, Vera Stadler, Dominik Baku, Florian Hennersdorf, Ulrike Ernemann, Ulf Ziemann, Sven Poli, Annerose Mengel

**Affiliations:** ^1^Department of Neurology & Stroke, Eberhard-Karls University of Tübingen, Tübingen, Germany; ^2^Centre for Neurovascular Diseases Tübingen, ZNET: Zentrum für neurovaskuläre Erkrankungen Tübingen, Tübingen, Germany; ^3^Department of Diagnostic & Interventional Neuroradiology, Eberhard-Karls University of Tübingen, Tübingen, Germany

**Keywords:** endovascular therapy, recanalization, acute ischemic stroke, neurovascular network, mechanical thrombectomy

## Abstract

**Background:** Interhospital transfer for endovascular treatment (EVT) within neurovascular networks might result in transfer of patients who will not undergo EVT (futile transfer). Limited evidence exists on factors associated with the primary patient selection for interhospital transfer from primary stroke centers (PSCs) to comprehensive stroke centers (CSCs), or EVT-workflow parameters that may render a transfer futile.

**Methods:** A prospective, registry-based study was performed between July 1, 2017 and June 30, 2018, at a hub-and-spoke neurovascular network in southwest Germany, comprising 12 referring PSCs and one designated CSC providing round-the-clock EVT at the University Hospital Tübingen. Patients with acute ischemic stroke due to suspected large artery occlusion (LAO) were included upon emergency interhospital transfer inquiry (ITI).

**Results:** ITI was made for 154 patients, 91 (59%) of whom were transferred to the CSC. Non-transferred patients (41%) had significantly higher premorbid modified Rankin scale scores (mRS) compared to transferred patients [median (IQR): 2 (1–3) vs. 0 (0–1), *p* < 0.001]. Interhospital transfer was denied due to: distal vessel occlusion (44.4%), or non-verifiable LAO (33.3%) in computed tomography angiography (CTA) upon teleconsultation by CSC neuroradiologists; limited Stroke-Unit or ventilation capacity (9.5%), or limited neuroradiological capacity at the CSC (12.7%). The CT-to-ITI interval was significantly longer in patients denied interhospital transfer [median (IQR): 43 (29–56) min] compared to transferred patients [29 (15–55), *p* = 0.029]. No further differences in EVT-workflow, and no differences in the 3-month mRS outcomes were noted between non-transferred and transferred patients [median (IQR): 2 (0–5) vs. 3 (1–4), *p* = 0.189]. After transfer to the CSC, 44 (48%) patients underwent EVT. The Alberta stroke program early CT score [ORadj (95% CI): 1.786 (1.573–2.028), *p* < 0.001] and the CT-to-ITI interval [0.994 (0.991–0.998), *p* = 0.001] were significant predictors of the likelihood of EVT performance.

**Conclusion:** Our findings show that hub-and-spoke neurovascular network infrastructures efficiently enable access to EVT to patients with AIS due to LAO, who are primarily admitted to PSCs without on-site EVT availability. As in real-world settings optimal allocation of EVT resources is warranted, teleconsultation by experienced endovascular interventionists and prompt interhospital-transfer-inquiries are crucial to reduce the futile transfer rates and optimize patient selection for EVT within neurovascular networks.

## Introduction

Endovascular recanalization therapy (EVT) has become standard of care in acute ischemic stroke (AIS) due to large artery occlusion (LAO) (1). The recent expansion of the therapeutic EVT time window, following the publication of the Clinical Mismatch in the Triage of Wake Up and Late Presenting Strokes Undergoing Neurointervention With Trevo (DAWN) (2) and the Endovascular Therapy Following Imaging Evaluation for Ischemic Stroke 3 (DEFUSE 3) (3) trial results, has led to a substantial increase in the number of EVT-eligible patients. In the face of rising healthcare demands, an optimal allocation of EVT resources—especially when EVT is provided within extended hub-and-spoke neurovascular networks—is warranted.

Currently, the operational workflow in most supraregional neurovascular networks (4, 5) entails acute AIS management, including administration of intravenous thrombolysis, at primary stroke centers (PSCs) followed by emergency interhospital patient transfer to comprehensive stroke centers (CSCs) when EVT is required. Although a growing number of studies have dealt with reasons for unsuccessful EVT in patients transferred from PSCs to CSCs (i.e., the “drip-and-ship” strategy) (6) compared to patients directly transferred to the nearest CSC (i.e., the “mothership” strategy) (7, 8), little is known regarding factors that determine the decision-making processes for patient-transfer within neurovascular networks. As current EVT registries, including the German Stroke Registry Endovascular Treatment (GSR-ET) (9, 10), only capture data of transferred patients with intention-to-treat with EVT at the CSCs, real-world evidence on patient selection for interhospital transfer are lacking.

Here we sought to identify factors associated with the primary patient selection following the request for emergency interhospital transfer for EVT within a large, supraregional neurovascular network. We aimed to evaluate the neurovascular network's capacity and operational workflow, and analyze factors associated with the decision to perform EVT, along with EVT and clinical outcomes in non-transferred and transferred patients. We hypothesized that data analyses of our prospective registry would yield results that contribute to quality improvement, enhancing the efficiency of decision-making and acute AIS care within the neurovascular network.

## Methods

Patient level data was acquired from prospective databases and transfer records from a hub-and-spoke neurovascular network in southwest Germany (Centre for neurovascular diseases Tübingen; “Zentrum für neurovaskuläre Erkrankungen Tübingen”: ZNET), comprising 12 referring PSCs and one designated CSC, that provides round-the-clock EVT at the University Hospital Tübingen. Consecutive patients presenting to PSCs, between July 1, 2017 and June 30, 2018, with AIS due to suspected LAO were included in the study upon emergency inter-hospital transfer inquiry (ITI) for EVT.

According to the ZNET standard operating procedures, and in line with operational protocols of supraregional neurovascular networks covering large rural and semi-rural areas (5), stroke patients were primarily admitted to the nearest PSC without bypassing hospitals by the ambulance service. Initial computed tomography (CT) imaging, including non-contrast CT (NCT) and CT angiography (CTA), was performed at the PSCs and eligible AIS patients underwent on-site intravenous thrombolysis. Adhering to the EVT guidelines in force at the time the study was conducted (1), ITI was made when a patient was considered eligible for EVT (with or without prior intravenous thrombolysis), according to the “drip-and-ship” paradigm. Eligibility criteria for ITI within the ZNET included: (a) anterior circulation LAO, within 6 h of symptom onset; (b) posterior circulation LAO, within 24 h of symptom onset; (c) any LAO in wake-up AIS or AIS with unknown symptom onset, and Alberta stroke program early CT score (ASPECTS) >6 (11). Non-accessible vessel occlusion was determined to include occlusions distal to the M2 segment, or any anterior or posterior cerebral artery segment. In the presence of contraindications for CTA that could cause significant delays in patient transfer (e.g., contrast agent allergy, potentially requiring patient stabilization after CTA), ITI could be made based on NCT when a patient presented with severe neurological deficits (12) and/or hyperdense artery sign on NCT (13). Evaluation of ITI followed on a case-by-case basis after assessment of clinical parameters, including premorbid modified Rankin scale (pmRS) score and National Institutes of Health Stroke Scale (NIHSS) score on admission. A telemedicine consultation was performed by a team of senior stroke neurologists and interventional neuroradiologists at the CSC, based on the real-time transmitted, cloud-based CT imaging data. If emergency patient transfer was decided, ambulance crew was recruited for air or road transport, on the principle of fastest-available-route for secondary transportation to the CSC. If emergency transfer within the ZNET was denied due to limited CSC Stroke Unit/Neurological ICU (NICU), ICU ventilation or neuroradiological (i.e., endovascular suite) capacity, ITI for emergency patient transfer to other neighboring hospitals with EVT availability was decided on individual basis. For patients transferred to the study CSC, CT imaging was performed on admission (o/a), including NCT, CTA, and CT perfusion (CTP) with cerebral blood flow (CBF) and cerebral blood volume (CBV) perfusion maps. Presence of concomitant vessel stenosis was diagnosed based on the North American Symptomatic Carotid Endarterectomy Trial (NASCET) criteria (14). CBF-CBV mismatch was visually assessed as described previously (2, 3). The final decision for EVT was made based on clinical and imaging findings by an interdisciplinary team of senior stroke neurologists and interventional neuroradiologists.

Based on the decisions to “ship” and perform EVT, 3 AIS patient groups were analyzed: (a) No-transfer group: Patients for whom ITI was made, but no transfer followed; (b) No-EVT group: transferred patients, who were considered unsuitable for EVT o/a to the CSC; (c) EVT group: transferred patients, who underwent EVT. Analysis was performed to determine clinical characteristics and process-related factors, including the time metrics: Symptom-onset-to-PSC-CT, Symptom-onset-to-intravenous-thrombolysis and PSC-CT-to-ITI, that could be associated with the decision to “ship.” For transferred patients, the time metrics PSC-CT-to-CSC-CT and CSC-CT-to-groin were also analyzed. Revascularization success was evaluated based on final angiograms. Successful recanalization was defined as modified treatment in cerebral infarction (mTICI) score ≥ 2b−3 for anterior circulation LAO (15) or Arterial Occlusive Lesion scale score = 3 for posterior circulation LAO (16). Clinical outcome was assessed by mRS at 90 days after the index event by phone calls or outpatient visits. If mRS 90 was not available, the mRS at discharge was carried forward.

The study was approved by the institutional ethics committee (Ethics Committee at the University Hospital of Tübingen, protocol number 767/2018BO2). Individual informed consent was waived for this study, since use of routine treatment data for research purposes is covered by a clinic-wide consent.

## Statistical Analyses

Differences between baseline variables in patient demographics and clinical characteristics were assessed using chi-square tests or two-tailed independent-sample Mann–Whitney *U* tests (due to non-normal distribution) depending on data characteristics, i.e., categorical vs. continuous variables, respectively. In the first part of the analysis, all observations were included. Patients for whom ITI was made, but no transfer followed comprised the “no-transfer group.” Transferred patients, who were unsuitable for EVT (“No-EVT group”) and patients, who underwent EVT (“EVT group”), jointly comprised the “transfer group.” Analysis was performed to determine clinical characteristics, imaging parameters and process-related factors associated with the decision to “ship.” A multiple regression analysis was conducted to assess the relationship between time metrics that were significantly different between transferred and non-transferred patients (i.e., PSC-CT-to-ITI time) and relevant patient characteristics (including age, pmRS, NIHSS o/a to the PSC, and presence of distal vessel occlusion).

In the second part of the analysis, differences in group characteristics were examined between the “No-EVT” and “EVT” patient groups. Analysis was performed to determine factors associated with the decision to perform EVT. A logistic regression analysis was used to calculate the odds of EVT performance including covariates significantly different at baseline or considered clinically relevant (i.e., PSC-CT-to-ITI time and ASPECTS). A Pearson product-moment correlation was run to determine the relationship between PSC-CT-to-ITI time and ASPECTS. We calculated the “Number-needed-to-ship” (NNS), defined as the number of patients needed to transfer to the CSC during the study period for one patient to undergo EVT (NNS=number of transferred patients/number of EVT patients).

In the third part of the analysis, EVT and clinical outcomes at 3 months (mRS) after the index event were analyzed as described previously. The significance level for all procedures was determined as *p* < 0.05. All statistical analyses were computed with IBM SPSS Statistics v.23 (IBM, NY, USA).

## Results

### Comparison of Patients Denied Interhospital Transfer (No-Transfer Group) vs. Patients Transferred to the CSC With Intention-to-Treat With EVT (Transfer Group)

During the study period, emergency ITI for EVT was made for a total of 154 patients, who presented with AIS due to suspected LAO in 12 referring PSCs. Of those, 63 patients (41%), who were denied transfer to the CSC comprised the “no-transfer group” vs. 91 patients (59%), who were transferred to the CSC with intention-to-treat with EVT and comprised the “transfer group.”

Patients' characteristics at baseline are depicted in Table 1.

**Table 1:** Baseline characteristics of non-transferred vs. transferred patients.

**Patient Characteristics**	**All (*n* = 154)**	**Non-transferred (*n* = 63)**	**Transferred (*n* = 91)**	***p*-Values**
Age, median (IQR)	77 (66–84)	80 (64–86)	75 (66–83)	0.199^§^
Female, n (%)	84 (54.5)	35 (55.6)	49 (53.8)	0.834^#^
**PSC Hospitals**				
Distance in km, median (IQR)	47 (18–61)	47 (18–61)	47 (18–61)	0.182^§^
**Baseline Parameters**				
NIHSS o/a, median (IQR)	11 (5–16)	9 (3–15)	13 (5–17)	0.074^§^
pmRS, median (IQR)	0 (0–2)	2 (1–3)	0 (0–1)	<0.001^*^^§^
ASPECTS at PSC, median (IQR)	10 (10–10)	10 (10–10)	10 (10–10)	0.378^§^
**Stroke time metrics and management**				
i.v. Thrombolysis, *n* (%)	84 (54.5)	21 (33.3)	63 (69.2)	<0.001^*^^#^
Onset-to-thrombolysis, median (IQR)	98 (72–133)	85 (73–131)	100 (71–135)	0.790^§^
Symptom-onset-to-CT in min, median (IQR)	69 (54–118)	72 (57–128)	67 (52–91)	0.115^§^
CTA within 15 min, *n* (%)	106 (68.8)	43 (68.3)	63 (69.2)	0.950^#^
PSC-CT-to-ITI in min, median (IQR)	37 (23–55)	43 (29–56)	29 (15–55)	0.029^*^^§^
ITI between 8 a.m. and 8 p.m., *n* (%)	65 (42.2)	25 (39.7)	40 (44)	0.893^#^
**Baseline CT imaging**				
Extracranial Stenosis NASCET above 70%, *n* (%)	10 (6.5)	2 (3.2)	8 (8.8)	0.164^#^
Extracranial Occlusion, *n* (%)	18 (11.7)	6 (9.5)	12 (13.2)	0.487^#^
Intracranial Occlusion, *n* (%)	119 (77.3)	36 (57.1)	83 (91.2)	<0.001^*^^#^
Cervical ICA Occlusion, *n* (%)	19 (12.3)	5 (7.9)	14 (15.4)	0.167^#^
Intracranial ICA Occlusion with Carotid-T, *n* (%)	7 (4.5)	1 (1.6)	6 (6.6)	0.148^#^
Intracranial ICA Occlusion without Carotid-T, *n* (%)	4 (2.6)	0 (0)	4 (4.4)	0.092^#^
Tandem occlusion ICA/MCA, *n* (%)	14 (9.1)	1 (1.6)	13 (14.3)	0.007^*^^#^
Proximal M1 Occlusion, *n* (%)	55 (35.7)	3 (4.8)	52 (57.1)	<0.001^*^^#^
Distal M1 Occlusion, *n* (%)	24 (15.6)	9 (14.3)	15 (16.5)	0.712^#^
M2 Occlusion, *n* (%)	31 (20.1)	18 (28.6)	13 (14.3)	0.030^*^^#^
Extracranial VA Occlusion, *n* (%)	1 (0.6)	0 (0)	1 (1.1)	0.404^#^
BA Occlusion, *n* (%)	9 (5.8)	1 (1.6)	8 (8.8)	0.061^#^
PCA Occlusion, *n* (%)	10 (6.5)	10 (15.9)	0 (0)	<0.001^*^^#^
Infratentorial Occlusion, *n* (%)	12 (7.8)	1 (1.6)	11 (12.1)	0.017^*^^#^
Vessel Tortuosity, *n* (%)	39 (25.3)	18 (28.6)	21 (23.1)	0.441^#^

§ *Mann–Whitney U tests*.

# *Chi-Square tests*.

* *Denotes significance p < 0.05*.

*IQR, interquartile range; PSC, Primary stroke center; NIHSS, National Institutes of Health Stroke Scale; pmRS, premorbid modified Rankin scale score; ASPECTS, Alberta stroke program early CT score; ITI, interhospital transfer inquiry; ICA, internal carotid artery; MCA, middle cerebral artery; VA, vertebral artery; BA, basilar artery; PCA, posterior cerebral artery*.

No statistically significant differences with respect to age, gender, or cardiovascular risk factors existed in non-transferred vs. transferred patients (Supplementary Material). Baseline NIHSS scores o/a to the PSCs were similar between groups, but non-transferred patients had significantly higher pmRS scores compared to transferred patients [median (IQR): 2 (1–3) vs. 0 (0–1), *p* < 0.001]. Significantly more transferred patients underwent intravenous thrombolysis compared to non-transferred patients (*p* < 0.001). The reasons for withholding intravenous thrombolysis included significantly higher rates of combined dual antiplatelet and direct oral anticoagulants (DOAC) in non-transferred patients (*p* = 0.031) and higher frequencies of demarcated ischemia o/a or admission outside the 4.5-h time window among non-transferred (25.4%) compared to transferred (13.2%) patients (*p* = 0.053) (Supplementary Material). In the subgroup of patients who underwent thrombolysis, the Symptom-onset-to-intravenous-thrombolysis time (in min) was similar between groups.

**Figure 1:**
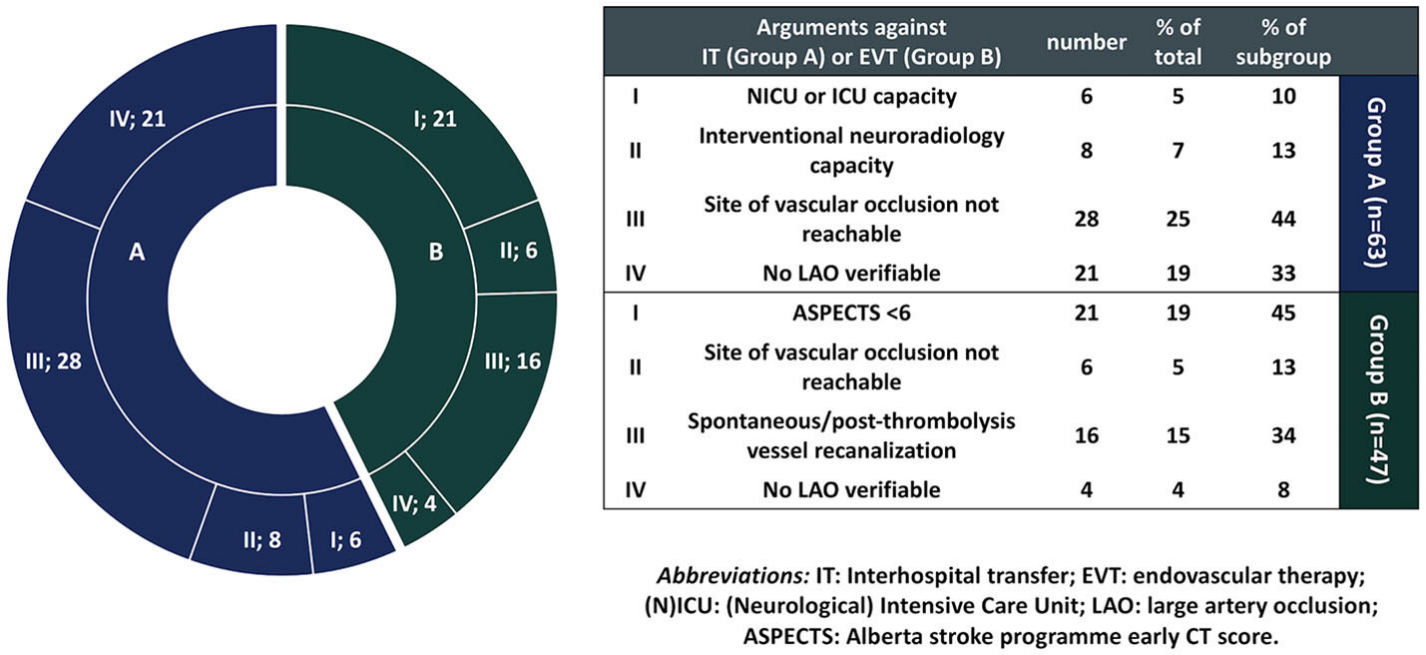
Reasons against interhospital patient transfer or endovascular therapy (EVT) in non-transferred and transferred patients who did not undergo EVT, respectively. In each diagram section, the number of patients is denoted next to the argument category for non-transferred (blue) or transferred no-EVT (green) patients, e.g., I; 6 denotes that point I (absent NICU or ICU capacity) was the reason against interhospital transfer (IT) in 6 non-transferred patients.

The reasons against interhospital transfer for the “no-transfer group” group are summarized in Figure 1. Six (9.5%) patients were denied interhospital transfer due to limited bed capacity at NICU or ICU ventilation capacity at the CSC, and 8 (12.7%) patients were denied transfer due to limited neuroradiological capacity. Upon teleconsultation, 28 (44.4%) patients were denied transfer due to non-accessible (i.e., distal) vessel occlusion. In 21 (33.3%) cases the suspicion of underlying LAO in CTA could not be verified by the CSC neuroradiologists on teleconsultation.

No significant between-group differences were noted with respect to the geographical distance (in km) between the PSCs and CSC, and no biases in patient selection were detected when the rates of non-transferred vs. transferred patients were compared for each referring PSC (Supplementary Material). Also, no between-group differences existed concerning the rates of non-transferred vs. transferred patients, based on whether ITI was made during (or outside) working hours (i.e., between 8:00 a.m. and 8:00 p.m.).

Regarding procedural parameters, PSC-CT-to-ITI time (in min) was significantly prolonged in non-transferred compared to transferred patients [median (IQR): 43 (29–56) vs. 29 (15–55), *p* = 0.029]. The equation of the multiple regression analyses, that assessed the relationship between PSC-CT-to-ITI time and patient characteristics, including age, pmRS, NIHSS o/a to the PSC, and presence of distal vessel occlusion, was significant *F*(4, 73) = 3.407, *p* = 0.013, with an *R*^2^ = 0.157. Advanced age (*b* = 0.251, *p* = 0.032) and higher pmRS (*b* = 0.229, *p* = 0.047) were significantly associated with prolonged PSC-CT-to-ITI time, while NIHSS o/a to the PSC (*b* = −0.002, *p* = 0.990) and distal vessel occlusion (*b* = 0.055, *p* = 0.628) were not related to the PSC-CT-to-ITI time. Symptom-onset-to-PSC-CT (in min) and ASPECT scores were comparable between groups. Although the rates of CTA performance were similar between groups, only 69% of all patients underwent CTA within 15 min after NCT.

With respect to the site of vessel occlusion, significantly more patients in the “transfer group” had intracranial occlusion (*p* < 0.001), tandem occlusion [i.e., internal carotid artery (ICA) and middle cerebral artery (MCA)] (*p* = 0.007), proximal M1 occlusion (*p* < 0.001) and infratentorial vessel occlusion (*p* = 0.017). Contrarily, significantly more patients from the “no-transfer group” had M2 occlusions (*p* = 0.03) and posterior cerebral artery (PCA) occlusions (*p* < 0.001).

### Comparison of Patients Considered Ineligible for EVT After Interhospital Transfer (No-EVT Group) vs. Patients Who Underwent EVT (EVT Group)

Among the 91 patients transferred to the CSC, 47 (52%) patients were considered ineligible for EVT and comprised the “No-EVT group” vs. 44 (48%) patients, who underwent EVT and comprised the “EVT group.” Accordingly, the NNS was 2 (=91 transferred patients/44 EVT patients). Patients' characteristics are depicted in Table 2.

**Table 2:** Baseline characteristics of No-EVT (ineligible for EVT) vs. EVT patients.

**Patient characteristics**	**All (*n* = 91)**	**No-EVT (*n* = 47)**	**EVT (*n* = 44)**	***p*-values**
Age, median (IQR)	75 (66–83)	74 (66–82)	76 (66–83)	0.812^§^
Female, *n* (%)	49 (53.8)	26 (55.3)	23 (52.3)	0.771^#^
**PSC Hospitals**				
Distance in km, median (IQR)	47 (18–61)	47 (33–61)	47 (18–61)	0.752^§^
Air-transportation, *n* (%)	16 (17.6)	8 (17.0)	8 (18.2)	0.884^#^
**Baseline Parameters**				
NIHSS at CSC, median (IQR)	5 (1–13)	6 (1–16)	5 (2–11)	0.659^§^
pmRS, median (IQR)	0 (0–1)	0 (0–1)	0 (0–0)	0.071^§^
ASPECTS at CSC, median (IQR)	9 (7–10)	7 (5–10)	10 (9–10)	0.001^*^^§^
**Stroke time metrics and management**				
i.v. Thrombolysis, *n* (%)	63 (69.2)	31 (66.0)	32 (72.7)	0.484^#^
Onset-to-thrombolysis, median (IQR)	100 (71–135)	100 (75–130)	90 (60–149)	0.577^§^
Symptom-onset-to-CT in min, median (IQR)	67 (52–91)	70 (54–83)	67 (46–143)	0.625^§^
CTA within 15 min, *n* (%)	63 (69.2)	32 (68.1)	31 (70.5)	0.679^#^
PSC-CT-to-ITI in min, median (IQR)	43 (24–65)	43 (30–86)	41 (19–59)	0.001^*^^§^
PSC-CT-to-CSC-CT in min, median (IQR)	125 (97–159)	125 (96–161)	125 (96–160)	0.704^§^
**CT Imaging at CSC**				
CTP Mismatch, *n* (%)	56 (61.5)	13 (27.7)	43 (97.7)	<0.001^*^^#^
Extracranial Stenosis NASCET above 70%, *n* (%)	8 (8.8)	3 (6.4)	5 (11.4)	0.402^#^
Extracranial Occlusion, *n* (%)	12 (13.2)	9 (19.1)	3 (6.8)	0.082^#^
Intracranial Occlusion, *n* (%)	83 (91.2)	39 (83.0)	44 (100)	0.004^*^^#^
Cervical ICA Occlusion, *n* (%)	14 (15.4)	11 (23.4)	3 (6.8)	0.028^*^^#^
Intracranial ICA Occlusion with Carotid-T, *n* (%)	6 (6.6)	5 (10.6)	1 (2.3)	0.108^#^
Intracranial ICA Occlusion without Carotid-T, *n* (%)	4 (4.4)	0 (0)	4 (9.1)	0.035^*^^#^
Tandem Occlusion ICA/MCA, *n* (%)	13 (14.3)	8 (17.0)	5 (11.4)	0.441^#^
Proximal M1 Occlusion, *n* (%)	52 (57.1)	27 (57.4)	25 (56.8)	0.952^#^
Distal M1 Occlusion, *n* (%)	15 (16.5)	5 (10.6)	10 (22.7)	0.120^#^
M2 Occlusion, *n* (%)	13 (14.3)	9 (19.1)	4 (9.1)	0.171^#^
Extracranial VA Occlusion, *n* (%)	1 (1.1)	1 (2.1)	0 (0)	0.331^#^
BA Occlusion, *n* (%)	8 (8.8)	5 (10.6)	3 (6.8)	0.520^#^
PCA Occlusion, *n* (%)	0 (0)	0 (0)	0 (0)	–
Infratentorial Occlusion, *n* (%)	11 (12.1)	8 (17.0)	3 (6.8)	0.136^#^
Vessel Tortuosity, *n* (%)	21 (23.1)	16 (34.0)	5 (11.4)	0.010^*^^#^

§ *Mann-Whitney U tests*;

# *Chi-Square tests*,

* *denotes significance p < 0.05*.

*IQR, interquartile range; PSC, Primary stroke center; NIHSS, National Institutes of Health Stroke Scale; CSC, Comprehensive stroke center; pmRS, premorbid modified Rankin scale score; ASPECTS, Alberta stroke program early CT score; ITI, interhospital transfer inquiry; CTP, CT perfusion; ICA, internal carotid artery; MCA, middle cerebral artery; VA, vertebral artery; BA, basilar artery; PCA, posterior cerebral artery*.

No significant between-group differences with respect to age, gender, cardiovascular risk factors and comorbidities, NIHSS o/a to the CSC and pmRS scores were noted (Supplementary Material). The rates of intravenous thrombolysis and the Symptom-onset-to-intravenous-thrombolysis time (in min) in patients who underwent thrombolysis were similar between No-EVT and EVT patients.

The reasons against EVT performance for the No-EVT group are summarized in Figure 1. Twenty-one (44.7%) patients were considered ineligible for EVT due to ASPECTS <6 o/a to the CSC, and 6 (12.8%) patients were considered ineligible for EVT due to distal vessel occlusion. In 16 (34%) cases no LAO existed o/a to the CSC, either due to spontaneous recanalization or recanalization following intravenous thrombolysis, and in 4 (8.5%) cases no LAO could be verified upon arrival at the CSC [in cases where no CTA had been performed at the PSCs and interhospital transfer was decided based on severity of neurological deficits—with NIHSS ≥ 14 (12) and/or hyperdense artery sign on NCT (13)].

No significant between-group differences were noted with respect to the geographical distance (in km) between the PSCs and CSC or the type of transportation (air or road transport). Also, no biases in patient selection were detected when the rates of EVT were compared for each referring PSC (Supplementary Material).

Regarding procedural parameters, PSC-CT-to-ITI time (in min) was significantly prolonged in the No-EVT compared to the EVT group [median (IQR): 43 (30–86) vs. 41 (19–59), *p* = 0.001]. Symptom-onset-to-PSC-CT (in min) and PSC-CT-to-CSC-CT (in min) were comparable between groups. Similar rates in CTA performance within 15 min after NCT (at the PSC) were observed between groups. Significantly higher ASPECT scores (at the CSC) were noted in the EVT compared to the No-EVT group [median (IQR): 10 (9–10) vs. 7 (5–10), *p* = 0.001]. The rates of CTP mismatch were significantly higher in the EVT compared to the No-EVT group (*p* < 0.001).

With respect to the site of vessel occlusion, significantly more patients in the EVT group had intracranial occlusion (*p* = 0.004) or intracranial ICA occlusion without occlusion of the carotid-T (*p* = 0.035). Contrarily, significantly more No-EVT patients had cervical ICA occlusions (*p* = 0.028) and vessel tortuosity in the CTA (*p* = 0.01).

### Predictors for Decision to Perform EVT After Patient Transfer to the CSC

The logistic regression model for assessment of the effect of ASPECT score o/a to the CSC and the PSC-CT-to-ITI time (in min) on the likelihood of EVT performance was statistically significant, χ^2^(2) =148.7, *p* < 0.001. The model explained 34.1% (Nagelkerke's *R*^2^) of the variance in EVT performance and correctly classified 71.6% of the cases (no multicollinearity was noted, tolerance = 0.946, VIF = 1.057). Higher ASPECT score [adjusted OR (ORadj) (95% CI): 1.786 (1.573–2.028), *p* < 0.001] was significantly associated with higher likelihood of EVT performance. Conversely, longer PSC-CT-to-ITI [0.994 (0.991–0.998), *p* = 0.001] was significantly associated with lower likelihood of EVT performance o/a to the CSC. A significant negative correlation was noted between PSC-CT-to-ITI time and ASPECTS o/a to the CSC (*r* = −0.233, *p* < 0.001).

### EVT Outcomes and Functional Outcome at 3 Months After AIS

Recanalization was achieved in 39 (88%) of EVT patients. Secondary intracerebral hemorrhage after EVT was noted in 7 (16%) patients. In terms of functional outcome at 90 days after the index event, after exclusion of patients with spontaneous recanalization o/a to the CSC, no significant differences were noted between No-EVT and EVT patients [median (IQR): 4 (2–5) vs. 3 (2–4), *p* = 0.138]. A subgroup analysis of patients with M1-occlusions revealed significant differences of functional outcome at 90 days, with No-EVT patients having higher mRS at 90 days compared to the EVT group [median (IQR): 5 (4–5) vs. 3 (2–4), *p* = 0.003]. No significant between-group differences in functional outcome at 90 days existed for the subgroups of patients with M2 occlusions (*p* = 0.352) and cervical ICA occlusions (*p* = 0.209). Also, no significant differences were noted in mRS outcome at 90 days between non-transferred and transferred patients [median (IQR): 2 (0–5) vs. 3 (1–4), *p* = 0.189].

## Discussion

We analyzed the operational workflow of EVT implementation and AIS service organization within a large, supraregional neurovascular network. Our findings indicate that the current infrastructures efficiently enable access to EVT for patients with AIS due to LAO, who are primarily admitted to PSCs without on-site EVT availability (5). During the study period, the majority (59%) of patients for whom emergency request for interhospital transfer was made were admitted to the CSC with intention-to-treat with EVT, 69.2% of whom underwent intravenous thrombolysis at the PSCs. Among non-transferred patients, interhospital transfer was denied due to inaccessible (i.e., distal) vessel occlusion or non-verifiable LAO upon teleconsultation in 44.4 and 33.3% of the cases, respectively. These results corroborate the role that PSCs hold in primary AIS care, including intravenous thrombolysis administration, and in candidate selection for EVT (4). Also, taking into consideration the finite EVT resources within high-volume neurovascular networks, these data emphasize the real-world significance of prompt teleconsultation by experienced interventionalists prior to interhospital patient transfers.

Since limited NICU/ICU or neuroradiological capacity at the CSC were the reasons against interhospital transfer in 9.5 and 12.7% of non-transferred patients, respectively, we investigated workflow parameters, including procedural time metrics, that could be optimized to improve resource allocation within the neurovascular network. Our results showed similar Symptom-onset-to-PSC-CT and Symptom-onset-to-intravenous-thrombolysis times between non-transferred and transferred patients. However, the PSC-CT-to-ITI times (i.e., the intervals spanning between CT performance and ITI) were significantly longer in patients denied interhospital transfer compared to transferred patients, while prolonged PSC-CT-to-ITI time was associated with advanced patient age and higher pmRS. These findings strongly suggest that, in clinical practice, workflow optimization may play a catalytic role in patient selection. In accord with previous real-world thrombectomy studies (4), our results underline the importance of early-on time-metrics monitoring, along with the need for regular training interventions (17) for workflow improvement starting from the stages of primary patient admission to the PSCs.

With respect to imaging studies, although no differences were noted in the CTA performance rates between non-transferred and transferred patients, only 69% of all patients admitted with AIS to the PSCs underwent CTA within 15 min of NCT. CTA delays comprise a detrimental, but modifiable factor when it comes to identification of patients that may benefit from EVT (18). Since multimodal CT imaging, including NCT, CTA and PCT, does not delay acute AIS management (19, 20), the integration of uniform AIS imaging protocols in the standard operating procedures of neurovascular networks is pivotal.

Within the study hub-and-spoke neurovascular network, we operationalized a quality assurance framework evaluating to what extent patient selection for interhospital transfer was aligned with contemporary EVT guidelines. In line with current EVT recommendations (21), non-transferred patients had higher premorbid mRS scores compared to transferred patients. Additionally, more non-transferred patients had distal (i.e., M2 and PCA) occlusions compared to transferred patients, whereas transferred patients had more frequently extracranial, vertebrobasilar, and proximal intracranial (i.e., M1) occlusions. Although these findings are in accordance with current EVT guidelines (1, 21), regular adjustment of patient selection criteria is warranted to ensure prompt translation of EVT research advances into clinical practice. Evidence from the MR CLEAN registry (Multicenter Randomized Clinical Trial of Endovascular Treatment of Acute Ischemic Stroke), for example, recently showed that prestroke-dependent patients can benefit from EVT to a similar extent as prestroke-independent patients (22). Additionally, EVT in distal vessel occlusion has recently become possible by use of modern EVT devices (23). Continuous adjustment of neurovascular networks' organization is, hence, imperative to facilitate up-to-date AIS care.

Comparing the interhospital transfer and EVT performance rates among transferred patients from the various referring PSCs, no biases in patient selection were detected. Although most EVT registries have, so far, neglected non-transferred patients (9), acquisition of pre-transfer data is crucial, especially in terms of assessment of PSCs' access to EVT and healthcare policy-making within neurovascular networks. Among transferred patients, the transportation times were comparable between patients excluded from EVT and EVT patients. These findings demonstrate that within the study neurovascular network timely access to EVT is facilitated for patients referred from the various PSCs.

During the study period, we found a NNS (“Number-needed-to-ship”) of 2, as 48% of transferred patients underwent EVT, whereas 52% were considered ineligible for EVT o/a to the CSC. In the majority (44.7%) of patients who were considered ineligible for EVT, ASPECTS was <6 o/a to the CSC, while absence of LAO o/a to the CSC or inaccessible vessel occlusion accounted for the remaining number of excluded cases. These results are comparable with data published from other EVT registries, reporting rates of futile transfers of 45% (6). We consider NNS an important measure for assessment of a neurovascular networks' efficiency in primary patient selection and performance across all stages of EVT implementation (i.e., with lower NNS indicating accurate primary patient selection at the PSCs, well-regulated interhospital transfer and patient selection for EVT at the CSC).

Consequently, we investigated factors associated with the NNS. Our analyses revealed that prolonged PSC-CT-to-ITI time is associated with significantly lower ASPECTS o/a to the CSC and significantly reduces the odds of EVT performance (i.e., thereby increasing the NNS). Conversely, higher ASPECTS was significantly associated with the decision to perform EVT. Since a large proportion of transferred patients (44.7%) were excluded from EVT o/a to the CSC due to ASPECTS <6, we hypothesize that adjunctive neuroprotective therapies, such as hyperbaric oxygenation (24), during primary care at the PSCs or during interhospital transfer could improve NNS. This hypothesis is currently being tested in a randomized clinical trial at the study CSC (ClinicalTrials.gov Identifier: NCT03500939).

Regarding EVT outcomes, successful recanalization was achieved in 88% of EVT patients, a rate that is within the upper range of rates reported from large EVT trials and registries (15, 25–27). Among transferred patients, patients who underwent EVT had a trend for better functional outcome at 3 months after the index event compared to patients excluded from EVT. Yet, this difference was significant only in the subgroup of patients presenting with M1 occlusion, favoring patients who underwent EVT. The 3-month functional outcomes in our study are also comparable to those obtained from studies evaluating the “drip-and-ship” over the “mothership” strategy (6), which suggest comparable clinical outcomes in patients undergoing mechanical thrombectomy according to the “drip-and-ship” or the “mothership” model (28), especially for PSCs located at considerable distance from the CSCs (29). Since at group level, however, the high recanalization rates in our study were not equally reflected in clinical outcome improvement, further research in patient selection for EVT is warranted. Finally, the comparable 3-month clinical outcomes between transferred and non-transferred patients support the efficiency of patient selection for interhospital transfer, i.e., with both patient groups receiving optimal care resulting in comparable outcomes despite the fact that transferred patients initially presented with more severe strokes in the presence of LAO. In non-transferred patients, underlying stroke etiologies other than LAO, including small vessel disease [i.e., which is associated with good functional outcome after stroke (30)], and distal vessel occlusions [i.e., which due to thrombus characteristics tend to respond to intravenous thrombolysis (31)] may further account for the comparable functional outcomes between groups.

### Limitations

We acknowledge possible limitations of the present study. First, as the present data were derived from a single neurovascular network with variable referral processes of the cooperating PSCs, additional real-world evidence is required to evaluate the generalizability of our results. Second, although the available time metrics reflect to a large extent the procedural stages involved in EVT implementation, monitoring of further time metrics [e.g., PSC-door-in-door-out and PSC-door-to-CSC-door times (4)] should be operationalized within neurovascular networks. Third, due to the limited number of patients in this study, data integration in predictive algorithms was not possible. Nonetheless, real-world data assimilation in predictive-decision models is crucial in order to improve patient selection, decision making over optimal transportation strategy (i.e., “drip-and-ship” over the “mothership”) and resource allocation within neurovascular networks (7).

## Conclusion

In conclusion, we provide evidence that the hub-and-spoke neurovascular network infrastructures efficiently enable access to EVT to patients with AIS due to LAO, who are primarily admitted to PSCs without on-site EVT availability. Our findings have important implications for neurovascular networks' service organization, as they point out that operational workflow should be monitored and optimized across all stages of EVT implementation, starting from the stages of primary admission to the PSCs. In particular, our findings show that delays in interhospital-transfer-inquiries can majorly compromise patient selection for interhospital transfer and render a transfer futile. Due to the crucial role of teleconsultation in real-world settings, major effort should be directed toward establishing uniform AIS imaging protocols across neurovascular networks, including multimodal CT imaging, to facilitate appropriate patient selection for EVT. Finally, regular auditing and training interventions are warranted to improve resource allocation within high-volume neurovascular networks.

## Data Availability Statement

The raw data supporting the conclusions of this article will be made available by the authors upon request.

## Ethics Statement

The studies involving human participants were reviewed and approved by Ethics Committee at the University Hospital of Tübingen. Written informed consent for participation was not required for this study in accordance with the national legislation and the institutional requirements.

## Author Contributions

AM and M-IS conceived the present study. AM, VS, DB, and M-IS acquired the data, analyzed/interpreted the data, and drafted the manuscript. FH and UE acquired the neuroradiological data and critically reviewed the manuscript. UZ and SP supervised and critically reviewed the manuscript for important intellectual content. All authors read and approved the final manuscript.

## Conflict of Interest

SP received speaker's honoraria and consulting honoraria from Bayer, Boehringer-Ingelheim, Bristol-Myers Squibb/Pfizer, Daiichi Sankyo and Werfen, reimbursement for congress traveling and accommodation from Bayer and Boehringer-Ingelheim, and research support from Bristol-Myers Squibb/Pfizer (significant), Boehringer-Ingelheim, Daiichi Sankyo (significant), and Helena Laboratories (all other contributions: modest). All competing interest are outside of the present work. UZ has received grants from European Research Council, German Research Foundation, German Ministry of Education and Research, Biogen Idec GmbH, Servier, and Janssen Pharmaceuticals NV, all not related to this work; and consulting honoraria from Biogen Idec GmbH, Bayer Vital GmbH, Bristol Myers Squibb GmbH, Pfizer, CorTec GmbH, Medtronic GmbH, all not related to this work. The remaining authors declare that the research was conducted in the absence of any commercial or financial relationships that could be construed as a potential conflict of interest.
