# Supplementary material for: Optimizing Patient Selection for Interhospital Transfer and Endovascular Therapy in Acute Ischemic Stroke: Real-World Data From a Supraregional, Hub-and-Spoke Neurovascular Network in Germany
Source: Front Neurol. 2020 Dec 4;11:600917. doi: 10.3389/fneur.2020.600917 (PMC7746873; doi:10.3389/fneur.2020.600917)
Supplement: Supplementary file 1 [file Data_Sheet_1.docx]

Supplementary Material

Supplementary Table 1. Baseline characteristics of non-transferred vs. transferred patients

^§^ Mann-Whitney U tests; ^#^ Chi-Square tests, *denotes significance p<0.05. *Abbreviations:* PSC: Primary stroke center, BMI: Body Mass Index, OAC: oral anticoagulant, DOAC: direct oral anticoagulant.

| **Patient Characteristics** | **All (n=154)** | | **Non-transferred (n=63)** | | **Transferred (n=91)** | | **p values** | |
| --- | --- | --- | --- | --- | --- | --- | --- | --- |
| **PSC Hospitals** |  | |  | |  | |  | |
| **Reutlingen, n (%)** | **46 (29.9)** | | **23 (36.5)** | | **23 (25.3)** | | **0.134^#^** | |
| **Albstadt, n (%)** | **39 (25.3)** | | **18 (28.6)** | | **21 (23.1)** | | **0.441^#^** | |
| **Freudenstadt, n (%)** | **13 (8.4)** | | **7 (11.1)** | | **6 (6.6)** | | **0.321^#^** | |
| **Kirchheim, n (%)** | **25 (16.2)** | | **6 (9.5)** | | **19 (20.9)** | | **0.060^#^** | |
| **Nagold, n (%)** | **5 (3.2)** | | **2 (3.2)** | | **3 (3.3)** | | **0.966^#^** | |
| **Calw, n (%)** | **9 (5.8)** | | **2 (3.2)** | | **7 (7.7)** | | **0.240^#^** | |
| **Rottweil, n (%)** | **6 (3.9)** | | **1 (1.6)** | | **5 (5.5)** | | **0.218^#^** | |
| **Sindelfingen, n (%)** | **3 (1.9)** | | **1 (1.6)** | | **2 (2.2)** | | **0.788^#^** | |
| **Sigmaringen, n (%)** | **3 (1.9)** | | **1 (1.6)** | | **2 (2.2)** | | **0.788^#^** | |
| **Friedrichshafen, n (%)** | **1 (0.6)** | | **1 (1.6)** | | **0 (0)** | | **0.228^#^** | |
| **Konstanz, n (%)** | **1 (0.6)** | | **1 (1.6)** | | **0 (0)** | | **0.228^#^** | |
| **Villingen-Schwenningen, n (%)** | **3 (1.9)** | | **0 (0)** | | **3 (3.3)** | | **0.146^#^** | |
| **Cardiovascular risk factors** | |  | | | | | | |
| **Hypertension, n (%)** | | **110 (71.4)** | | **45 (71.4)** | | **65 (71.4)** | | **0.706^#^** |
| **Atrial fibrillation, n (%)** | | **67 (43.5)** | | **23 (36.5)** | | **44 (48.4)** | | **0.235^#^** |
| **Diabetes mellitus, n (%)** | | **34 (22.1)** | | **13 (20.6)** | | **21 (23.1)** | | **0.783^#^** |
| **Obesity (BMI > 30), n (%)** | | **28 (18.2)** | | **12 (19)** | | **16 (17.6)** | | **0.757^#^** |
| **Smoking, n (%)** | | **23 (14.9)** | | **8 (12.7)** | | **15 (16.5)** | | **0.560^#^** |
| **Coronary artery disease, n (%)** | | **29 (18.8)** | | **14 (22.2)** | | **15 (16.5)** | | **0.327^#^** |
| **Peripheral artery disease, n (%)** | | **6 (3.9)** | | **2 (3.2)** | | **4 (4.4)** | | **0.724^#^** |
| **Secondary prophylaxis at baseline** | | | | | | | |  |
| **None, n (%)** | | **73 (47.4)** | | **21 (33.3)** | | **52 (57.1)** | | **0.008*^#^** |
| **OAC, n (%)** | | **12 (7.8)** | | **6 (9.5)** | | **6 (6.6)** | | **0.442^#^** |
| **DOAC, n (%)** | | **21 (13.6)** | | **10 (15.9)** | | **11 (12.1)** | | **0.417^#^** |
| **Antiplatelet monotherapy, n (%)** | | **37 (24)** | | **17 (27.0)** | | **20 (22)** | | **0.362^#^** |
| **Dual antiplatelet therapy, n (%)** | | **3 (1.9)** | | **2 (3.2)** | | **1 (1.1)** | | **0.333^#^** |
| **Dual antiplatelet therapy and DOAC, n (%)** | | **3 (1.9)** | | **3 (4.8)** | | **0 (0)** | | **0.031*^#^** |
| **Reason against thrombolysis** | |  | |  | |  | |  |
| **Demarcated ischemia or outside the time window, n (%)** | | **28 (18.2)** | | **16 (25.4)** | | **12 (13.2)** | | **0.053^#^** |
| **OAC, n (%)** | | **7 (4.5)** | | **3 (4.8)** | | **4 (4.4)** | | **0.915^#^** |
| **DOAC, n (%)** | | **5 (3.2)** | | **1 (1.6)** | | **4 (4.4)** | | **0.334^#^** |
| **Other, n (%)** | | **13 (8.4)** | | **8 (12.7)** | | **5 (5.5)** | | **0.114^#^** |

Supplementary Table 2. Baseline characteristics of No-EVT (ineligible for EVT) vs. EVT patients

^§^ Mann-Whitney U tests; ^#^ Chi-Square tests, *denotes significance p<0.05. *Abbreviations:* PSC: Primary stroke center, BMI: Body Mass Index, OAC: oral anticoagulant, DOAC: direct oral anticoagulant.

| **Patient Characteristics** | **All (n=91)** | **No-EVT (n=47)** | **EVT (n=44)** | **p values** |
| --- | --- | --- | --- | --- |
| **PSC Hospitals** | | | | |
| **Reutlingen, n (%)** | **23 (25.3)** | **10 (21.3)** | **13 (29.5)** | **0.364^#^** |
| **Albstadt, n (%)** | **21 (23.1)** | **12 (25.5)** | **9 (20.5)** | **0.566^#^** |
| **Freudenstadt, n (%)** | **6 (6.6)** | **3 (6.4)** | **3 (6.8)** | **0.933^#^** |
| **Kirchheim, n (%)** | **19 (20.9)** | **10 (21.3)** | **9 (20.5)** | **0.923^#^** |
| **Nagold, n (%)** | **3 (3.3)** | **2 (4.3)** | **1 (2.3)** | **0.597^#^** |
| **Calw, n (%)** | **7 (7.7)** | **3 (6.4)** | **4 (9.1)** | **0.628^#^** |
| **Rottweil, n (%)** | **5 (5.5)** | **2 (4.3)** | **3 (6.8)** | **0.592^#^** |
| **Sindelfingen, n (%)** | **2 (2.2)** | **2 (4.3)** | **0 (0)** | **0.166^#^** |
| **Sigmaringen, n (%)** | **2 (2.2)** | **1 (2.1)** | **1 (2.3)** | **0.962^#^** |
| **Friedrichshafen, n (%)** | **0 (0)** | **0 (0)** | **0 (0)** | **-** |
| **Konstanz, n (%)** | **0 (0)** | **0 (0)** | **0 (0)** | **-** |
| **Villingen-Schwenningen, n (%)** | **3 (3.3)** | **2 (4.3)** | **1 (2.3)** | **0.597^#^** |
| **Cardiovascular risk factors** | | | | |
| **Hypertension, n (%)** | **65 (71.4)** | **34 (72.3)** | **31 (70.5)** | **0.714^#^** |
| **Atrial fibrillation, n (%)** | **44 (48.4)** | **21 (44.7)** | **23 (52.3)** | **0.530^#^** |
| **Diabetes mellitus, n (%)** | **21 (23.1)** | **11 (23.4)** | **10 (22.7)** | **0.942^#^** |
| **Obesity (BMI > 30), n (%)** | **16 (17.6)** | **9 (19.1)** | **7 (15.9)** | **0.687^#^** |
| **Smoking, n (%)** | **15 (16.5)** | **8 (17.0)** | **7 (15.9)** | **0.889^#^** |
| **Coronary artery disease, n (%)** | **15 (16.5)** | **6 (12.8)** | **9 (20.5)** | **0.321^#^** |
| **Peripheral artery disease, n (%)** | **4 (4.4)** | **1 (2.1)** | **3 (6.8)** | **0.274^#^** |
| **Secondary prophylaxis at baseline** |  |  |  |  |
| **None, n (%)** | **52 (57.1)** | **27 (57.4)** | **25 (56.8)** | **0.947^#^** |
| **OAC, n (%)** | **6 (6.6)** | **3 (6.4)** | **3 (6.8)** | **0.910^#^** |
| **DOAC, n (%)** | **11 (12.1)** | **8 (17.0)** | **3 (6.8)** | **0.146^#^** |
| **Antiplatelet monotherapy, n (%)** | **20 (22.0)** | **8 (17.0)** | **12 (27.3)** | **0.215^#^** |
| **Dual antiplatelet therapy, n (%)** | **1 (1.1)** | **1 (2.1)** | **1 (2.3)** | **0.336^#^** |
| **Dual antiplatelet therapy and DOAC, n (%)** | **0 (0)** | **0 (0)** | **0 (0)** | **-** |
